# UPLC-QTOF-MS Based Comparison of Rotundic Acid Metabolic Profiles in Normal and NAFLD Rats

**DOI:** 10.3390/metabo13010038

**Published:** 2022-12-26

**Authors:** Lvying Wu, Lei Xing, Yake Zou, Zichen Wang, Yuanyuan Gou, Lei Zhang, Su Guan

**Affiliations:** 1 MOE Joint International Research Laboratory of Synthetic Biology and Medicine, School of Biology and Biological Engineering, South China University of Technology, Guangzhou 510006, China; 2 Guangdong Provincial Key Laboratory of New Drug Design and Evaluation, Guangzhou 510407, China

**Keywords:** metabolite, NAFLD, rats, rotundic acid, UPLC-QTOF-MS

## Abstract

Rotundic acid, the principal bioactive constituent of the herbal remedy “Jiubiying”, has been considered as a candidate compound for treating non-alcoholic fatty liver disease (NAFLD). However, the in vivo and in vitro metabolism of rotundic acid has remained unclear. With the aim of elucidating its metabolic profile, a reliable approach that used ultra-high performance liquid chromatography combined with quadrupole time-of-flight mass spectrometry (UPLC-QTOF-MS) was applied for screening and identifying rotundic acid in vivo (plasma, feces, urine, and liver tissue of normal and NAFLD model rats) and in vitro (rat liver microsomes) metabolites. Herein, 26 metabolites of rotundic acid were identified, including 22 metabolites in normal rats, 20 metabolites in NAFLD model rats, and eight metabolites in rat liver microsomes. Among them, 17 metabolites were identified for the first time. These data illustrate that the pathological status of NAFLD affects the metabolism of rotundic acid. Furthermore, the major pathways of metabolism included phase Ⅰ (demethylation, desaturation, etc.) and phase Ⅱ (sulfation and glucuronidation) reactions, as well as a combined multiple-step metabolism. This work provides important information on the metabolism of rotundic acid and lays the foundation for its future clinical application.

## 1. Introduction

Non-alcoholic fatty liver disease (NAFLD), featured by liver macrovesicular steatosis caused by factors other than excessive alcohol use, is a chronic liver disease that affects people worldwide [1,2]. NAFLD is highly correlated with obesity, cardiovascular disease, and insulin resistance, and is a vital predisposing element for cirrhosis and hepatocellular carcinoma pathogenesis [3]. The latest data shows that over 25% of the population suffers from NAFLD globally, and the incidence is gradually growing year after year [4]. Due to its uncertain pathogenesis, no effective drugs have been approved by the Food and Drug Administration (FDA) for treating NAFLD [5]. Hence, it is critical to develop safe and effective drugs to treat NAFLD.

Natural products are an excellent source modern drug development. Between 1981 and 2019, almost 70% of drugs approved by the FDA were natural products or corresponding derivatives [6]. “Jiubiying” is the dry leaf and bark of Ilex rotunda Thumb, which is often used to treat diarrhea, metaphysitis, bruises, colds, fever, and rheumatism [7,8]. Furthermore, it is currently included in the Chinese Pharmacopoeia. Rotundic acid (RA) is the main bioactive component in “Jiubiying” and belongs to the pentacyclic triterpenoids [9]. Increasing reports have demonstrated that Rotundic acid (RA) possesses many pharmacological functions, including, but not limited to, anti-cancer and anti-inflammatory activities [10,11,12,13]. Additionally, it has been revealed that RA can prevent and alleviate hepatic disorders [12,14,15]. Yuan-Man Hsu and co-workers [12] have found that RA has a significant lipid-lowering effect, with mild anti-inflammatory activity in diabetic mice, while reducing liver lipid droplets. Our previous study, which aimed to evaluate the pharmacological effect and mechanism of action of RA on NAFLD, illustrated that RA effectively alleviated hepatic lipid accumulation in the NAFLD rat model [16]. These findings make RA a promising candidate compound for treating NAFLD.

Drug metabolism plays a vital role in explaining and predicting efficacy and toxicity [17,18]. Hence, a thorough study of metabolic fate becomes an integral part of drug discovery. So far, the information on RA metabolism is limited to only one study, and only 11 metabolites of RA have been identified in normal rats [19]. The comprehensive metabolic pathways of RA have not been fully elucidated. Moreover, because of its important role in drug metabolism, changes in liver function under pathological states, such as NAFLD, are bound to affect certain enzymes and transporters which are related to the internal metabolism and transportation of drugs. It was reported that RA is a substrate of P-glycoprotein and can be substantially metabolized by Cytochrome P450 (CYP) 3A4 [20], which explains its relatively low oral bioavailability in rats (16–19%) [21]. Moreover, our previous study into the pharmacological effect of RA on NAFLD showed that RA had no effect on the oleic acid-induced rat primary hepatocyte in vitro model, but effectively ameliorated non-alcoholic steatohepatitis after oral administration in rats [16]. This suggests that the effect in the rats may be attributed to the metabolites of RA in vivo, other than its prototype. Thus, it is necessary to study the metabolism of RA in NAFLD. In this study, we induced a rat NAFLD model by feeding animals with a high-fat diet. Next, we employed the ultra-performance liquid chromatography combined a with quadrupole time-of-flight tandem mass spectrometry (UPLC-QTOF-MS) approach under automatic MS^E^ mode to determine RA metabolites both in normal and NAFLD rats, as well as in rat liver microsomes, which helped us to propose the pathways of metabolism of RA underlying normal and pathological states.

## 2. Materials and Methods

### 2.1. Material and Chemicals

The reference standard of RA (purity > 98%) was purchased from the Nanjing Spring & Autumn Biotech Co., Ltd (Nanjing, China). Liver microsomes of SD rat (20 mg/mL) were purchased from the Research Institute for Liver Diseases Co., Ltd. (Shanghai, China). Ethyl acetate, methanol, and acetonitrile (Fisher Scientific, Hampton, NH, USA) were all HPLC grade, and formic acid (Fluka, Radnor, PA, USA) was LC-MS grade. A Milli-Q system (Billerica USA) was utilized to prepare the deionized water. The high-fat diet (HFD) encompassed 78% primary feed, 10% egg yolk powder, 10% lard, 2% cholesterol, and 0.5% sodium cholate, and was acquired from the Trophic Animal Feed High-Tech CO., Ltd. (Nantong, China). The colorimetric assay kits for malondialdehyde (MDA) and superoxide dismutase (SOD) were bought from the Nanjing Jiancheng Bioengineering Institute (Nanjing, China).

### 2.2. Animals Experiments

#### 2.2.1. Modeling of NAFLD Rats

The Committee of Ethics of Animal Experimentation of Guangdong Pharmaceutical University approved the animal experiments (Ref:2017226). All animal experiments were accomplished in rigorous accordance with the Guide for the Care and Use of Laboratory Animals.

Sprague-Dawley rats (male and 180–200 g) were supplied by the Laboratory Animal Center of the Guangzhou University of Chinese Medicine (Guangzhou, China) and kept in environmentally controlled circumstances including a 12 h light/12 h dark cycle and a stable room temperature (25 ± 2 °C). The NAFLD rats model was induced using a 5-week high-fat diet according to the literature [1,22] and our preliminary experiments. Meanwhile, rats in the normal group were fed a regular diet during the experimental period. After overnight fasting, RA (60 mg/kg prepared in 0.5% CMC-Na) was administrated orally to both normal and NAFLD rats. Metabolic cages were utilized to maintain the rats for urine and feces collection within 0–24 h. Blood samples were obtained at 30 min, 2 h, 6 h, 12 h and 24 h, and were spun at 5000 rpm for 10 min to separate the plasma. After 24 h blood sampling, liver tissue samples were obtained under anesthesia to analyze metabolites and confirm the success of NAFLD modeling. The blank plasma, urine, feces, and liver tissue were collected in the same procedure described above after vehicle treatment. The biological samples were kept at −80 °C.

#### 2.2.2. Pathological Staining

Liver samples were secured in paraformaldehyde (4%) and addressed for dehydration, paraffin embedding, and were sectioned (4 μm). The sections were then deparaffinized and stained with hematoxylin and eosin. An upright optical microscope (Nikon, NIKON ECLIPSE E100) was employed to observe the results.

Frozen liver sections, which were stained with Oil Red solution (Servicebio G1016) for 10 min and immersed in hematoxylin (Servicebio G1004) for counterstaining for 5 min, were observed under a microscope (LEICA DM 4000 B LED). The lipid accumulation area was analyzed by Image-Pro Plus 6.0 by evaluating integrated optical density (IOD).

The above staining procedure referred to the reported method with some modification [23]. The histological evaluation of liver slices was random.

#### 2.2.3. Biochemical Detection

According to the proportion of 1:9 (*W*/*V*), a 0.2 g liver sample was added into ice-cold 0.9% NaCl, ground to make a 10% homogenate, and spun for 10 min at 4 °C (5000 rpm). The supernatant was then gathered. The concentration of hepatic cholesterol (CHOL) and triglycerides (TG) was determined by an automatic biochemical analyzer (Hitachi 7180, Hitachi High-Tech Corp., Ibaraki, Japan). The serum SOD and MDA were determined by the commercial kits under the instruction of the manufacturer.

### 2.3. Preparations of Biological Samples

Referring to our previously published method with a slight modification, the preparation of biological samples was executed [17].

- Plasma

The plasma samples, which were obtained from individual rats, were mixed in equal amounts into 200 μL, and then treated to precipitate proteins with 600 μL acetonitrile. The procedure included centrifugation (12,000 rpm, 10 min), the collection of supernatants, drying at room temperature by a vacuum concentrator (Eppendorf, Hamburg, Germany), and the reconstitution of the residue in 100 μL diluent containing methanol and water (1:1, *v*/*v*).

- Urine

A 200 μL urine sample was precipitated with 400 μL methanol and mixed thoroughly. The other procedure was the same as that of plasma samples.

- Feces

The freeze-dried feces were ground into powder. Next, 4 mL methanol was used to immerse the fecal powder (0.4 g), and the metabolites in the solution underwent a 30-min extraction by ultrasound. After centrifugation (8000 rpm, 10 min), the collected upper layer was dried by evaporation at room temperature. A 200 μL diluent containing a mixture (1:1, *v*/*v*) of methanol and water was then employed to reconstitute the residue.

- Liver tissues

One gram of liver sample and 1 mL of 0.9% sodium chloride solution were mixed and thoroughly ground. In order to precipitate proteins in the liver sample mixture, the acetonitrile was added to the liver sample at a ratio of 4:1, then vortexed for 3 min. The rest of the procedure was similar to the method used for feces, except that the volume of reconstituted solution was 300 μL.

Prior to UPLC-QTOF-MS analysis, all samples were spun for 10 min at 4 °C (12,000 rpm).

### 2.4. RA Metabolism In Vitro

For in vitro metabolism, according to a previous report [24], RA (200 ng/mL, dissolved in methanol) was incubated in rat liver microsomal incubation solution (200 μL) consisting of hepatic microsomes [1.0 mg (protein)/mL], phosphate-buffered saline (0.1 M PBS, pH 7.4) and nicotinamide adenine dinucleotide phosphate (NADPH, 1 mM). The mixture of rat hepatic microsomes, phosphate-buffered saline, and RA was preincubated at 37 °C for 5 min before the addition of 1 mM NADPH. After that, the system was incubated at 37 °C for 30 min to address the reaction. Next, 1 mL ice-cold ethyl acetate solution was added to bring the reaction to an end. The collection and drying of the upper organic layer were conducted by centrifugation (12,000 rpm, 10 min) and evaporation, respectively. Thereafter, 50 μL acetonitrile-water solution (1:1, *v*/*v*) was applied to dissolve the residue. The blank control samples were prepared without RA. After centrifugation (12,000 rpm, 10 min), 5 μL supernatant was subjected to analysis. The incubation was conducted in triplicate.

### 2.5. UPLC-QTOF-MS Conditions

Based on our previously instrumental methodology [17], a UPLC system (Waters ACQUITY) fitted with a reverse-phase UPLC BEH C18 column (Waters Acquity, 100 × 2.1 mm^2^, i.d. 1.7 μm) with an in-line filter at 40 °C was utilized to chromatographically separate the metabolites. A linear gradient elution consisting of mobile phase A (0.1% formic acid) and mobile phase B (acetonitrile) was employed: maintained 5% B at 0–2 min, 5–50% B at 2–7 min, maintained 50% B at 7–11 min, 50–85% B at 11–18 min, maintained 85% B at 18–20 min, 85–5% B at 20–20.5 min and held for 2 min. The velocity of flow and the run time were 0.4 mL/min and 22.5 min for each sample, respectively. The injection volume was 5 µL.

A Waters Q-TOF SYNAPT G2 Spectrometer equipped with ESI source under negative ion mode was utilized to conduct mass spectrometry detection, for which the full scan mode was set at the mass range of 100–1200 Da. The superlative parameters of MS for maximum sensitivity were set: the cone voltage was 30 V and capillary voltage was 3.0 kV; the desolvation temperature was 550 °C and source temperature was 120 °C; the desolvation gas (N_2_) flow rate was 700 L/h and the cone gas flow rate was 50 L/h. In MS^E^ centroid mode, the MS data were acquired with the low energy function in the trap collision energy (6 eV), and the tandem mass data were acquired with the high energy function in the ramp trap collision energy (20–50 eV). During MS analysis, in order to acquire accurate mass, the Leucine enkephalin was used as the lock mass of *m*/*z* 554.2615 ([M-H]^−^). The instrument operation and data acquisition were monitored by Masslynx NT 4.1 (Waters, Milford, MA, USA).

### 2.6. Data Analysis

The Metabolynx XS software (Waters, MA, USA) with the mass defect filter (MDF) was utilized to process the metabolism data. The MDF window was ±0.1 Da, 5 ppm was used as the maximum tolerance for mass error, and the spectrum was 2% higher than the relative intensity. The data of pathological and biochemical changes are presented as mean ± standard deviation (SD), and were analyzed by Student’s *t*-test. *p* < 0.05 was considered as statistically significant.

## 3. Results

### 3.1. Establishment of NAFLD Model

**Figure 1:**
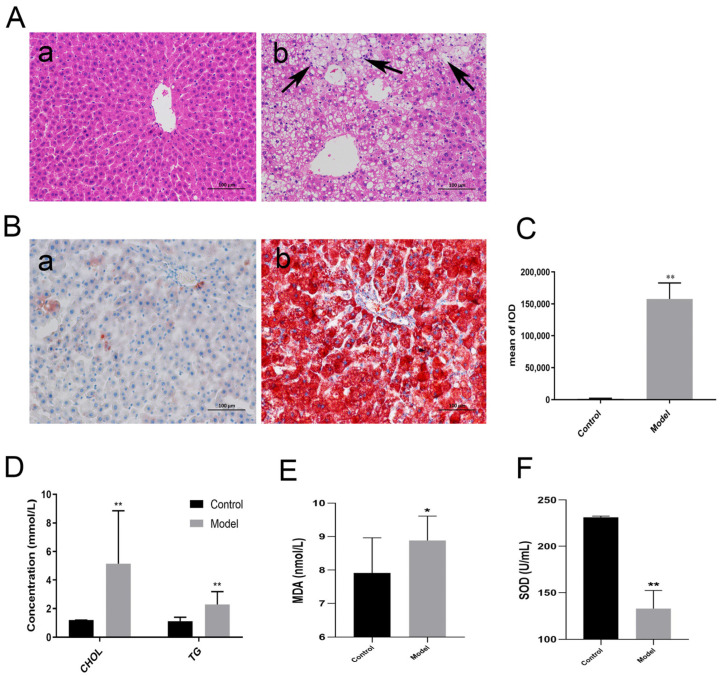
Establishment of NAFLD model. (**A**) The histopathological examination by H&E (200×). (**a**): Normal control group; (**b**): NAFLD model group. The black arrows indicate lipid droplets and inflammatory infiltrate. (**B**) Oil red o (×200) staining in control (**a**) and model (**b**) groups. (**C**) Quantitative analysis of oil red staining. IOD: integral optical density. (**D**) The level of CHOL and TG in the liver. (**E**) The concentration of MDA in serum. (**F**) The concentration of SOD in serum. Data are exhibited as the mean ± SD (n = 10/group). * indicates *p* < 0.05, ** indicates *p* < 0.01.

The liver pathological sections were evaluated by H&E stain, a “gold standard” for the diagnosis of NAFLD, which provided reliable evidence on the establishment of the NAFLD model. As illustrated in Figure 1A, significant microvesicular steatosis and the inflammatory changes of hepatic lobules were observed in the NAFLD group when compared to the normal group, which is the typical NAFLD feature. This suggested the success of the establishment of the NAFLD rat model in the current study. Similarly, in the oil red staining (Figure 1B,C), high-fat diet feeding resulted in an elevated area of lipid accumulation and aggravated steatosis in the model group, while there was no significant change in the control group.

Matching the pathological outcomes, the concentrations of the hepatic CHOL and TG (Figure 1D) in the model groups was obviously higher than those in the control groups. Malondialdehyde (MDA) is the main product of lipid peroxidation in the body, while superoxide dismutase (SOD) exerts its function to improve the oxidative stress state of the body and inhibit lipid peroxidation. As indicated in Figure 1E,F, the level of MDA in the NAFLD group was substantially increased, whereas the content of SOD reduced, indicating that lipid metabolism in the body was disordered.

### 3.2. The Characteristic Fragmentation of RA

It is confirmed that metabolites and parent compounds share the same splitting properties. Thus, the analysis of the fragmentation characteristics of RA is helpful and crucial to deduce and recognize RA (M0) and its metabolites [17,25]. In this study, the RA standard was assessed under both positive and negative modes of the ESI source. RA gave a higher signal intensity under the negative mode. In addition, RA was eluted at 8.04 min under the analysis conditions, and the deprotonated mass [M-H]^−^ was 487.3420 (C_30_H_47_O_5_^−^).

**Figure 2:**
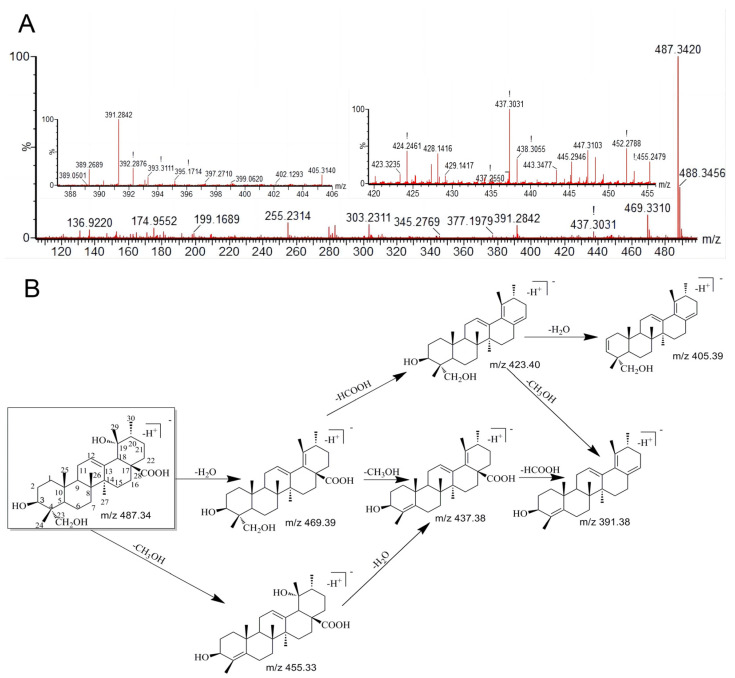
The MS^2^ spectra of RA (**A**) and the proposed fragmentation process of RA (**B**). “!”indicates there may be interference with the shape or resolution of the particular MS peaks. It usually appears on the magnified Masslynx mass specta. For known chemical structures, these molecular ions can be manually determined.

The incorporation of the MS^2^ fragment information with the previous reports [26,27,28] and the fragment ions of RA observed were mostly constituted by the continuing losses of neutral molecules, involving CO_2,_ which is 44 Da, CH_4_O, which is 32 Da, H_2_O, which is 18 Da, and HCOOH, which is 46 Da. In its MS/MS fragmentation pattern (Figure 2A), RA provided ample fragment ions at *m*/*z* 469.3310 produced by a reduction of H_2_O (18 Da) and at *m*/*z* 437.3031 constituted through the successive neutral cleavages of H_2_O and CH_4_O (32 Da). Furthermore, the daughter product at *m*/*z* 455.2479 was formed by the fragmentation of CH_4_O, *m*/*z* 423.3235 was generated by the consecutive losses of H_2_O and HCOOH, *m*/*z* 405.3140 was via the successive eliminations of H_2_O, HCOOH, and H_2_O, *m*/*z* 393.3111 was obtained by the consecutive cleavages of H_2_O, CH_4_O, and CO_2_, and *m*/*z* 391.3871 was formed through the successive losses of H_2_O, HCOOH, and CH_4_O. The detailed formation pathway of RA fragmentation ions, which is based on the structural properties and MS/MS fragment ions, is proposed in Figure 2.

### 3.3. Identification of the Metabolites of RA

**Figure 3:**
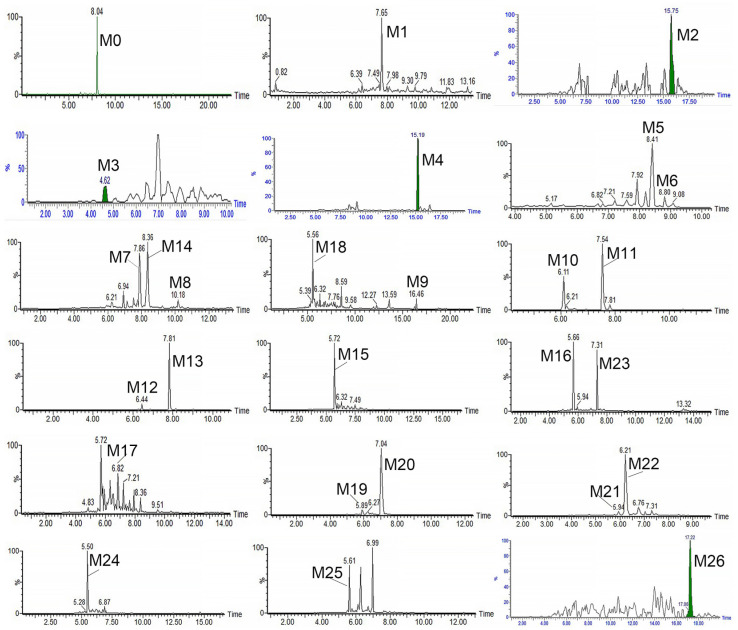
The extracted ion chromatograms of RA and its metabolites.

In this study, compared with blank samples, parent compound RA (M0) and its 26 metabolites were determined both in vivo and in vitro, and were identified by accurate mass, elemental compositions, MS/MS fragment information, and reference literature information. An overview of the characteristics of all metabolites is listed (Table 1). The total ion chromatograms are shown in Figure S1. The extracted ion chromatograms of the metabolites are presented (Figure 3), and the MS^2^ spectra of the metabolites are displayed in Figure S2.

Metabolite M1 (t_R_ = 7.65 min) showed the [M-H]^−^ ion at *m*/*z* 473.3260 (C_29_H_46_O_5_). Compared with M0, the reduced 14 Da mass change indicated the cleavage of CH_2_ from M0. In its MS/MS spectrum, the daughter ions of M1 at *m*/*z* 455.2859, 423.2159 were formed by the continuous eliminations of H_2_O and CH_4_O, respectively. Furthermore, the product ions at *m*/*z* 409.2301, 405.2641, 391.2842 were generated by further losses of H_2_O and HCOOH. The fragmentation behavior of M1 was similar to that of RA. Based on this evidence and the literature [19], M1 was appraised as the demethylation metabolite of M0.

M2 (t_R_ = 15.75 min), which exhibited the [M-H]^−^ ion at *m*/*z* 445.3348 (C_28_H_46_O_4_), was 42 Da (with the cleavages of CH_2_ and CO) lighter than M0. The primary fragmentation ions at 427.35 and *m*/*z* 395.35 in its MS/MS spectrum matched the consecutively neutral cleavages of H_2_O and CH_4_O. Meanwhile, the product ions at *m*/*z* 427.35 and 395.35 were 28 Da less than the typical product ions of M1 (*m*/*z* 455.2859 and 423.2159), suggesting that M2 was formed by the decarbonylation of M1.

M3 (t_R_ = 4.62 min) showed the [M-H]^−^ ion at *m*/*z* 649.3563 (C_35_H_54_O_11_) and was 176 Da heavier than the prototype of M1 and 162 Da heavier than the prototype of RA. The daughter ions were at 631.21 [M-H-H_2_O]^−^, 599.20 [M-H-H_2_O-CH_4_O]^−^ and 423.20 [M-H-H_2_O-CH_4_O-glcUA]^−^ in its MS/MS spectrum, which indicated that M3 corresponded to the glucuronide conjugate of M1.

M4 (t_R_ = 15.19 min) displayed the deprotonated [M-H]^−^ ion at *m*/*z* 621.3605 (C_34_H_54_O_10_). It was 176 Da (the addition of C_6_H_8_O_6_) heavier than M2. The fragmentation ions at *m*/*z* 603.43 [M-H-H_2_O]^−^, 553.31 [M-H-2H_2_O-CH_4_O]^−^, 445.39 [M-H-glcUA]^−^, and 395.34 [M-H-H_2_O-CH_4_O-glcUA]^−^, suggested that M4 was the glucuronide conjugate of M2.

M5 (t_R_ = 8.41 min) and M6 (t_R_ = 8.80 min), with 2 Da less than M0, exhibited the same deprotonated molecular [M-H]^−^ ion at *m*/*z* 485.3267 (C_30_H_46_O_5_). In their MS/MS spectra, M5 and M6 displayed the same product ion at *m*/*z* 467.3573 [M-H-H_2_O]^−^, which was also 2 Da lighter than the product ion (*m*/*z* 469.3310) of RA. Moreover, the representative fragmentation ions of M5 were at *m*/*z* 455.3588 [M-H-2CH_3_]^−^, 437.3543 [M-H-2CH_3_-H_2_O]^−^, 411.3806 [M-H-2CH_3_-CO_2_]^−^, and 389.3309 [M-H-H_2_O-HCOOH-CH_4_O]^−^. According to the tandem mass behavior and the previous reports [19,27], M5 could be confirmed as rotundanonic acid. The daughter ion at *m*/*z* 405.3729, discovered in the MS/MS spectrum of M6, was consistent with [M-H-2H_2_O-CO_2_]^−^. Finally, according to the above results and the literature [19], M5 and M6 were speculated to be the isomers of the dehydrogenation metabolites of RA.

M7 (t_R_ = 7.86 min) and M8 (t_R_ = 10.18 min), which was 14 Da (with the addition of CH_2_) heavier that of M5 and M6, displayed the same deprotonated formula of C_31_H_48_O_5_ ([M-H]^−^, *m*/*z* 499.3423). The product ions, *m*/*z* 481.3273 [M-H-H_2_O]^−^, 469.3383 [M-H-2CH_3_]^−^, and 419.3471 [M-H-2H_2_O-CO_2_]^−^, were also 14 Da heavier than the corresponding product ions of M5 and M6 (*m*/*z* 467.3573, 455.3588, 405.3729),which indicated the presence of methylation. Other fragmentation ions, *m*/*z* 485.3659 [M-H-CH_2_]^−^ and 439.3662 [M-H-CH_2_-HCOOH]^−^, were found in their MS/MS spectra as well. Hence, M7 and M8 were tentatively elucidated as the methylation products of dehydrogenation M0.

M9 (t_R_ = 16.46min) presented a [M-H]^−^ ion at *m*/*z* 661.3616 (C_36_H_54_O_11_) and was 176 Da (the addition of C_6_H_8_O_6_) heavier than M5 and M6. The daughter ions at *m*/*z* 643.39 [M-H-H_2_O]^−^ and 581.44 [M-H-2H_2_O-CO_2_]^−^ were 176 Da heavier than the corresponding daughter ions of M6 (*m*/*z* 467.3567 and 405.3729). This suggested that M9 was the glucuronide conjugate of dehydrogenated M0.

M10 (t_R_ = 6.11 min) and M11 (t_R_ = 7.54 min) displayed the identical molecular ion [M-H]^−^ at *m*/*z* 503.3372 (C_30_H_48_O_6_). The 16 Da mass addition suggested an oxygen atom introduction of M0 (503 − 487 = 16). In their MS/MS spectra, the product ions at *m*/*z* 485.3713 (through the elimination of an H_2_O), 453.3587 (through the losses of H_2_O and CH_4_O), and 407.3573 (through the sequentially neutral cleavages of H_2_O, CH_4_O and HCOOH) were shown. The tandem mass behaviors were similar to M0. Thus, it was proposed that M10 and M11 were the isomers of hydroxylation products of RA. The literature [19] supports this speculation.

M12 (t_R_ = 6.44 min) and M13 (t_R_ = 7.81 min), which displayed the identical deprotonated molecule of C_30_H_46_O_6_ ([M-H]^−^, *m*/*z* 501.3216), were 14 Da heavier than M0, indicating one oxygen atom addition and two hydrogen atoms loss of M0 (501 − 487 = 14). The product ions of M12 and M13 shown at *m*/*z* 483.3675 [M-H-H_2_O]^−^, 469.3797 [M-H-CH_4_O]^−^, 451.2440 [M-H-H_2_O-CH_4_O]^−^ and 405.3373 [M-H-H_2_O-CH_4_O-HCOOH]^−^, were 2 Da less than the product ions at *m*/*z* 485.3713, 471.3570, 453.3587 and 407.3552 of M10 and M11, suggesting the occurrence of dehydrogenation. Accordingly, M12 and M13 were diagnosed as the isomers of hydroxylated and dehydrogenated products of RA.

M14 (t_R_ = 8.36 min) presented the [M-H]^−^ ion at *m*/*z* 499.3107 (C_30_H_44_O_6_). It was 2 Da lighter than M12 and M13. The fragmentation ions at *m*/*z* 481.3325 [M-H-H_2_O]^−^ and 449.3637[M-H-H_2_O-CH_4_O]^−^, was also 2 Da lighter than the fragmentation ions of M12 (*m*/*z* 483.3675, 451.2440), indicating that M14 was the dehydrogenation product of M12.

M15 (t_R_ = 5.72 min) displayed the deprotonated molecule of C_29_H_46_O_6_. Compared with M0, the [M-H]^−^ ion of M15 at *m*/*z* 489.3261 was 2 Da (489 − 487 = 2) heavier than that of RA, indicating an oxygen atom addition and a methyl group cleavage of RA’s molecular formula. The primary daughter ions exhibited at *m*/*z* 471.3112 [M-H-H_2_O]^−^, 439.2938 [M-H-H_2_O-CH_4_O]^−^ and 421.2827 [M-H-2H_2_O-CH_4_O]^−^, were also 2 Da heavier than the correlated product ions of RA. As a consequence, M15 was determined as the demethylation and oxidation metabolites of RA, which was also reported in Li’s work [19].

M16 (t_R_ = 5.66 min, *m*/*z* 519.3369), which exhibited the molecular formula of C_30_H_48_O_7_, consisted of the introduction of two oxygen atoms (32 Da) of RA. The product ions at *m*/*z* 501.2282 [M-H-H_2_O]^−^, 469.2262 [M-H-H_2_O-CH_4_O]^−^ and 423.3068 [M-H-H_2_O-CH_4_O-HCOOH]^−^ found in its MS/MS spectrum, corresponding to the sequential cleavages of H_2_O, CH_4_O, and HCOOH, were 32 Da heavier than those of RA. Thus, M16 was proposed as the di-hydroxylated product of RA. This metabolite was detected in normal rats reported by Li et al. [19]. However, we only detected it in NAFLD rats.

M17(t_R_ = 6.82 min) showed the deprotonated molecular [M-H]^−^ ion at *m*/*z* 489.3506 (C_30_H_50_O_5_). It was 2 Da (with the addition of two hydrogens) heavier than M0. The main fragmentation ions were at *m*/*z* 471.2795 [M-H-H_2_O]^−^ and 407.3333 [M-H-2H_2_O-HCOOH]^−^. Thus, M17 was determined as the reduction of M0.

M18 (t_R_ = 5.56 min) displayed the deprotonated formula of C_30_H_50_O_6_ ([M-H]^−^, *m*/*z* 505.3398). It was 16 Da (the addition of one oxygen) heavier than the molecular weight of M17. The [M-H]^−^ ion gave rise to the daughter ions at *m*/*z* 487.3348 [M-H-H_2_O]^−^, 423.3238 [M-H-2H_2_O-HCOOH]^−^ and 391.2398 [M-H-2H_2_O-HCOOH-CH_4_O]^−^, indicating that M18 was the hydroxylation of M17.

M19 (t_R_ = 5.89 min) and M20 (t_R_ = 7.04 min) displayed the identical deprotonated formula of C_30_H_48_O_8_S ([M-H]^−^, *m*/*z* 567.2737), which were 80 Da (with the introduction of SO_3_) higher than the molecular weight of RA. In their MS/MS spectra, M19 showed fragmentation ions at *m*/*z* 549.3577 [M-H-H_2_O]^−^, 503.2623 [M-H-H_2_O-HCOOH]^−^, 489.3547 [M-H-HCOOH-CH_4_O]^−^, and 485.2561 [M-H-2H_2_O-HCOOH]^−^, which were similar to the fragmentation behaviors of M0. However, the position of sulfate conjugation for M19 remained inconclusive. In comparison, M20 showed fragmentation ions at *m*/*z* 523.3328 [M-H-CO_2_]^−^, 487.3748 [M-H-SO_3_]^−^, 453.2393 [M-H-2H_2_O-CH_4_O-HCOOH]^−^ and 407.3369 [M-H-2H_2_O-CO_2_-SO_3_]^−^, which were consistent with the previous report [25,28,29], suggesting that sulfate conjugation presented at C-3. Thus, M19 and M20 were tentatively elucidated as the sulfate conjugate product of RA.

M21 (t_R_ = 5.94 min) and M22 (t_R_ = 6.21 min) shared the same molecular formula of C_29_H_46_O_8_S with the deprotonated ion at *m*/*z* 553.2967. The product ion at 473.2652 [M-H-SO_3_]^−^, indicated that M21 and M22 corresponded to the sulfate conjugate of M1. Meanwhile, other product ions such as *m*/*z* 503.3317 [M-H-H_2_O-CH_4_O]^−^, 489.3580 [M-H-H_2_O-HCOOH]^−^, 485.2623 [M-H-2H_2_O-CH_4_O]^−^, 429.2460 [M-H-CO_2_-SO_3_]^−^ and 379.3122 [M-H-H_2_O-CH_4_O-CO_2_-SO_3_]^−^ were also found. Thus, M21 and M22 were diagnosed as the sulfate conjugate and demethylated products of RA.

M23 (t_R_ = 7.31 min) showed the deprotonated ion at *m*/*z* 565.2891 (C_30_H_46_O_8_S). It was 80 Da (the addition of SO_3_, 565 − 485 = 80) heavier than the molecular weight of M5 and M6 (485.3267). Furthermore, it also gave rise to daughter ions at *m*/*z* 515.3238 [M-H-H_2_O-CH_4_O]^−^, 501.3277 [M-H-H_2_O-HCOOH]^−^, 485.3311 [M-H-SO_3_]^−^, 439.3272 [M-H-HCOOH-SO_3_]^−^, and 421.3105 [M-H-H_2_O-HCOOH-SO_3_]^−^. Similar to M20, it was speculated that the sulfate conjugation occurred at C-3 based on the previous report [25,28]. Therefore, M23 was proposed as the sulfate conjugate product of dehydrogenated RA.

M24 (t_R_ = 5.50 min) was characterized as the deprotonated quasi-molecular ion at *m*/*z* 583.2958 ([M-H]^−^, C_30_H_48_O_9_S), which was 80 Da (the addition of SO_3_, 583 − 503 = 80) heavier than that at *m*/*z* 503.3372 (M10, M11). Meanwhile, it also generated product ions at *m*/*z* 565.2947 [M-H-H_2_O]^−^, 533.3004 [M-H-H_2_O-CH_4_O]^−^, 467.2841 [M-H-2H_2_O-SO_3_]^−^, and 439.3002 [M-H-H_2_O-HCOOH-SO_3_]^−^. Accordingly, M24 was proposed to be the sulfate conjugate product of hydroxylated RA.

M25 (t_R_ = 5.61 min) was characterized with the [M-H]^−^ ion at *m*/*z* 581.2805(C_30_H_46_O_9_S), which was 80 Da (581 − 501 = 80) heavier than M12 and M13. The daughter ions at *m*/*z* 563.2995 [M-H-H_2_O]^−^, 517.2929 [M-H-H_2_O-HCOOH-SO_3_]^−^, 501.2476 [M-H-SO_3_]^−^, 487.2725 [M-H-H_2_O-CH_4_O-CO_2_]^−^, and 467.2502[M-H-2H_2_O-CH_4_O-HCOOH]^−^ were showed in its MS/MS spectrum. Thus, M25 was identified to be the sulfate conjugate of M12 or M13.

M26 (t_R_ = 17.22 min) displayed the prototype formula of C_36_H_56_O_11_ with the [M-H]^−^ ion at *m*/*z* 663.3741. It was 176 Da (with the introduction of glcUA) heavier than the molecular weight of M0. The daughter ions of M26 showed at *m*/*z* 549.22 [M-H-2H_2_O-CH_4_O-HCOOH]^−^, 487.22 [M-H-glcUA]^−^, and 437.31 [M-H-H_2_O-CH_4_O-glcUA]. Therefore, M26 was diagnosed as the C-3 glucuronide conjugate product of RA, which is consistent with a previous study [19].

**Figure 4:**
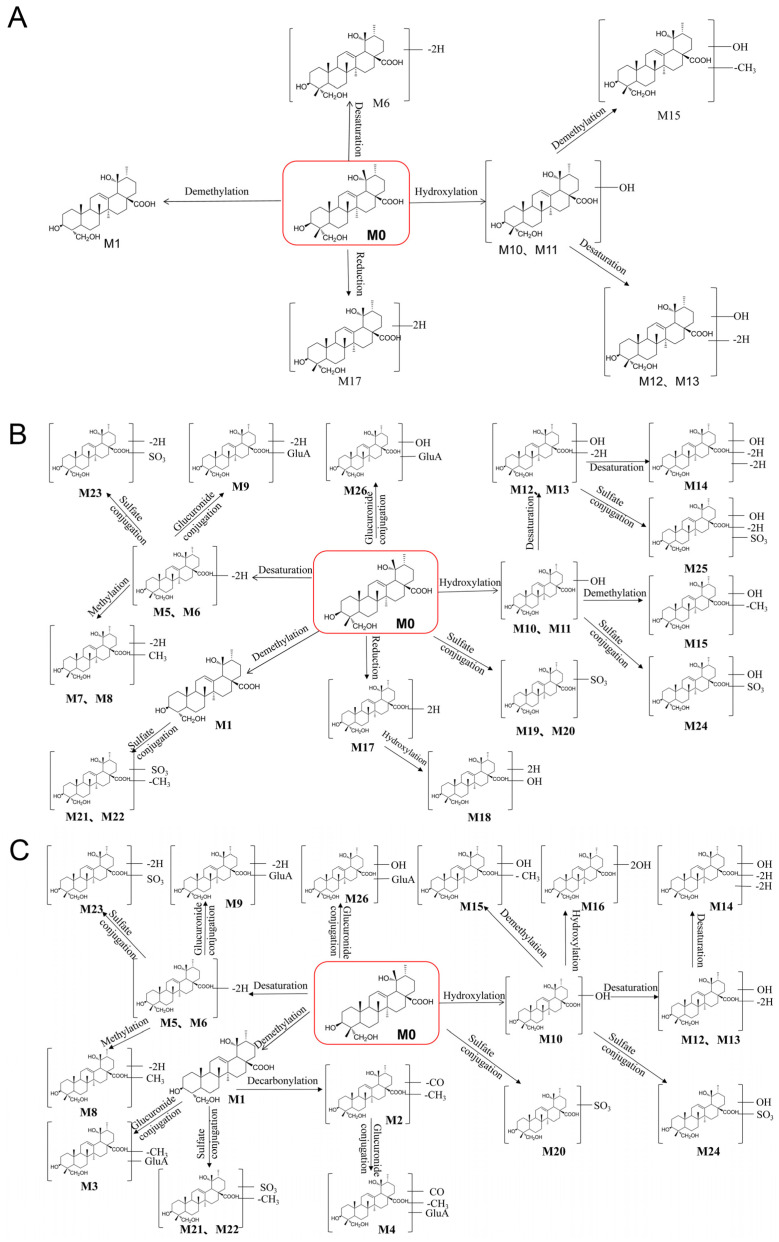
The proposed metabolic profiles of RA, in rat liver microsome (**A**), in normal rat (**B**), and in NAFLD rat (**C**).

In summary, 26 metabolites were detected and identified, including eight metabolites in vitro, and 26 metabolites in vivo. Among them, compared with previous reports [19], 17 metabolites (except for M1, M5, M6, M10, M11, M15, M16, M20, M26) were detected for the first time. According to the above analyses, the possible metabolic profiles for RA in vivo (normal and NAFLD rats) and in vitro rat liver microsome incubation were proposed (Figure 4). As shown in Figure 4, RA underwent extensive metabolism including phase Ⅰ reactions (desaturation, demethylation, reduction, and hydroxylation), phase Ⅱ reactions (methylation, sulfation, and glucuronidation), as well as multiple-step metabolism, which may explain the low bioavailability of RA. However, the exact structure of the metabolites needs to be confirmed by further study due to the lack of standards.

## 4. Discussion

The in vitro metabolism of RA was performed in a rat liver microsome incubation system. Rat liver microsomes are an excellent model in vitro for drug metabolism due to the low cost, high-throughput and high efficiency [29]. The cytochrome P450 enzymes (Phase I reactions) markedly expressed in rat liver microsome offer predictive value for in vivo drug metabolism. In the current study, eight metabolites of RA (M1, M6, M10–M13, M15, M17) were detected in rat liver microsomes, all of which were also found in the in vivo metabolism of RA in normal rats. However, this conclusion is limited by the lack of phase II reactions in the liver microsome. This requires further detailed study.

Furthermore, after the oral administration of RA, 22 metabolites were detected in normal rat samples, including 22 in feces, 12 in plasma, 14 in urine, and 13 in the liver. To better understand the influence of NAFLD’s pathological status on in vivo RA metabolism, the metabolic profile in NAFLD rats was conducted and compared with that in normal rats. In total, 20 metabolites were detected in NAFLD rat samples, comprising 16 in feces, 9 in plasma, 12 in urine, and 12 in the liver. From these results, obvious differences were observed in normal and NAFLD model rats. Six metabolites (M7, M11, M17–M19, M25) were only detected in the normal rats, while four metabolites (M2–M4, M16) were only detected in NAFLD model rat samples. There were fewer classes of metabolites determined in NAFLD rats, and the reduction of RA only occurred in normal rats. It was reported that NAFLD’s physiological status could affect the quantity and function of hepatic drug metabolism enzymes [30]. In this study, the liver suffered some damage in NAFLD rats. Therefore, the divergent metabolism between normal and NAFLD rats was possibly owing to changes in drug metabolism enzymes under the pathological condition. In this study, after the oral administration of RA, most metabolites were detected in feces, which was consistent with Li’s report [19], suggesting that feces are the main metabolic clearance way of RA and its metabolites. In addition, according to previous reports [31,32,33], the gut microbiome may change dramatically in NAFLD rats. Thus, the alteration of the gut microbiome in NAFLD rats was proposed as another reason for the metabolic differences. The speculation that the altered liver function and gut microbiome led to the changed metabolic profile of RA was supported by the fact that in the current study, the feces of normal rats contained all metabolites, while the feces of NAFLD rats lacked some metabolites.

## 5. Conclusions

In conclusion, a comprehensive metabolic profile of RA in vivo and in vitro was elucidated utilizing UPLC-Q/TOF-MS. Taken together, 26 metabolites were determined, including 22 metabolites in normal rats, 20 metabolites in NAFLD rats, and eight metabolites in vitro. Among them, 17 metabolites were identified for the first time. The major metabolic reactions of RA included demethylation, desaturation, hydroxylation, reduction, sulfation, and glucuronidation. There are differences regarding the metabolite types between the normal and NAFLD model rats, which suggested that the pathological status of NAFLD may affect the RA metabolism. This study offers reliable scientific evidence for a comprehensive understanding of the mechanism of RA regarding efficacy and side effects, which will eventually benefit the clinical application of RA.

## Figures and Tables

**Table 1:** The metabolite information of RA.

NO.	t_R_ (Min)	[M-H]^−^		Formula	Metabolite Description	MS/MS Fragment	Normal Rat				NAFLD Rat				LM
		Cal.	Exp.				F	P	U	L	F	P	U	L	
0	8.04	487.3423	487.3420	C_30_H_48_O_5_	Prototype	469.3310, 455.2479, 437.3031, 423.3235, 405.3140, 393.3111, 391.2842	+	+	+	+	+	+	+	+	+
1	7.65	473.3267	473.3260	C_29_H_46_O_5_	Demethylation	455.2859, 423.2159, 409.2301, 405.2641, 391.2842	+	+	+	+	+	+	+	−	+
2	15.75	445.3317	445.3348	C_28_H_46_O_4_	Demethylation + Decarbonylation	427.35, 395.35, 391.34	−	−	−	−	−	−	+	+	−
3	4.62	649.3587	649.3563	C_35_H_54_O_11_	Demethylation + Glucuronide conjugation	631.21, 599.20, 423.20	−	−	−	−	+	+	+	−	−
4	15.19	621.3638	621.3605	C_34_H_54_O_10_	Demethylation + Decarboxylation + Glucuronide conjugation	603.43, 553.31, 445.39, 395.34	−	−	−	−	−	+	−	+	−
5	8.41	485.3267	485.3317	C_30_H_46_O_5_	Dehydrogenation	467.3573, 455.3588, 437.3543, 411.3806, 389.3309	+	+	+	+	+	−	+	+	−
6	8.8	485.3267	485.3273	C_30_H_46_O_5_	Dehydrogenation	467.3567, 405.3729, 389.3342	+	+	−	−	+	+	+	−	+
7	7.86	499.3423	499.3423	C_31_H_48_O_5_	Desaturation + Methylation	485.3569, 481.3273, 469.3383, 419.3471, 437.3595	+	+	+	−	−	−	−	−	−
8	10.18	499.3423	499.3358	C_31_H_48_O_5_	Desaturation + Methylation	481.3222, 439.3662	+	−	−	+	+	−	−	+	−
9	16.46	661.3587	661.3616	C_36_H_54_O_11_	Desaturation + Glucuronide conjugation	643.39, 581.44, 409.24	+	+	−	+	+	+	+	+	−
10	6.11	503.3372	503.3366	C_30_H_48_O_6_	Hydroxylation	485.3713, 453.3587, 407.3573, 391.3818	+	−	+	−	+	+	−	+	+
11	7.54	503.3372	503.3387	C_30_H_48_O_6_	Hydroxylation	485.3718, 471.3510, 453.3677, 407.3552, 391.3692	+	+	+	+	−	−	−	−	+
12	6.44	501.3216	501.3211	C_30_H_46_O_6_	Hydroxylation + desaturation	483.3675, 439.3691, 451.2440, 405.3373	+	+	−	−	+	−	+	−	+
13	7.81	501.3216	501.3580	C_30_H_46_O_6_	Hydroxylation + desaturation	483.3567, 469.3797, 439.3788, 421.3737, 405.3845	+	+	+	+	+	−	+	−	+
14	8.36	499.3059	499.3107	C_30_H_44_O_6_	Hydroxylation + desaturation + Desaturation	481.3325, 457.2943, 449.3637	+	−	+	+	+	−	−	−	−
15	5.72	489.3216	489.3261	C_29_H_46_O_6_	Hydroxylation + Demethylation	471.3112, 439.2938, 421.2827	+	−	+	+	+	+	+	+	+
16	5.66	519.3321	519.3369	C_30_H_48_O_7_	2 x Hydroxylation	501.2282, 469.2262, 423.3068	−	−	−	−	+	−	−	+	−
17	6.82	489.358	489.3506	C_30_H_50_O_5_	Reduction	471.2795, 407.3333	+	−	−	+	−	−	−	−	+
18	5.56	505.3529	505.3398	C_30_H_50_O_6_	Reduction + Hydroxylation	487.3348, 423.3238, 391.2398	+	−	+	−	−	−	−	−	−
19	5.89	567.2991	567.2737	C_30_H_48_O_8_S	Sulfate conjugation	549.3577, 503.2623, 489.3547, 485.2561	+	−	−	−	−	−	−	−	−
20	7.04	567.2991	567.3094	C_30_H_48_O_8_S	Sulfate conjugation	523.3328, 487.3748, 453.2393, 407.3369	+	+	+	−	+	−	−	+	−
21	5.94	553.2835	553.3008	C_29_H_46_O_8_S	Demethylation + Sulfate conjugation	485.2623, 489.3580, 473.2652, 429.2460, 379.2833	+	+	+	+	−	−	−	+	−
22	6.21	553.2835	553.2967	C_29_H_46_O_8_S	Demethylation + Sulfate conjugation	503.3317, 489.3503, 485.2567, 473.3644, 429.2452, 379.3122	+	+	−	+	−	+	−	+	−
23	7.31	565.2835	565.2891	C_30_H_46_O_8_S	Desaturation + Sulfate conjugation	515.3238, 501.3277, 485.3311, 439.3272, 421.3105	+	−	−	+	+	+	+	−	−
24	5.5	583.294	583.2958	C_30_H_48_O_9_S	Hydroxylation + Sulfate conjugation	565.2947, 533.3004, 467.2841, 439.3002	+	−	+	+	+	−	+	−	−
25	5.61	581.2784	581.2805	C_30_H_46_O_9_S	Hydroxylation + desaturation + Sulfate conjugation	563.2995, 517.2929, 501.2476, 487.2725, 467.2502	+	−	+	−	−	−	−	−	−
26	17.22	663.3744	663.3741	C_36_H_56_O_11_	Glucuronide conjugation	549.22, 487.22, 437.31	+	+	+	−	+	−	+	+	−

T_R_, retention time; F, feces; P, plasma; U, urine; L, liver; LM, liver microsome.

## Data Availability

The data submitted in this study are available within this article and in the Supplementary Materials.

## Supplementary Materials

The following supporting information can be downloaded at: https://www.mdpi.com/article/10.3390/metabo13010038/s1, Figure S1: Typical chromatograms of rat plasma, urine, fecal and liver, as well as liver microsomal samples. N–F: Fecal sample from normal rats; M–F: Fecal sample from model rats; N–P: Plasma sample from normal rats; M–P: Plasma sample from model rats; N–U: Urine sample from normal rats; M–U: Urine sample from model rats; N–L: Liver sample from normal rats; M–L: Liver sample from model rats; LM: Liver microsomal samples; Figure S2: The MS/MS spectra of metabolites M1–M26.
