# Supplementary material for: UPLC-QTOF-MS Based Comparison of Rotundic Acid Metabolic Profiles in Normal and NAFLD Rats
Source: Metabolites. 2022 Dec 26;13(1):38. doi: 10.3390/metabo13010038 (PMC9861526; doi:10.3390/metabo13010038)
Supplement: Supplementary file 1 [file metabolites-13-00038-s001.zip › metabolites-2053701-supplementary.pdf]

Supplementary materials

UPLC-QTOF-MS based comparison of Rotundic acid metabolic profiles in normal and NAFLD rats

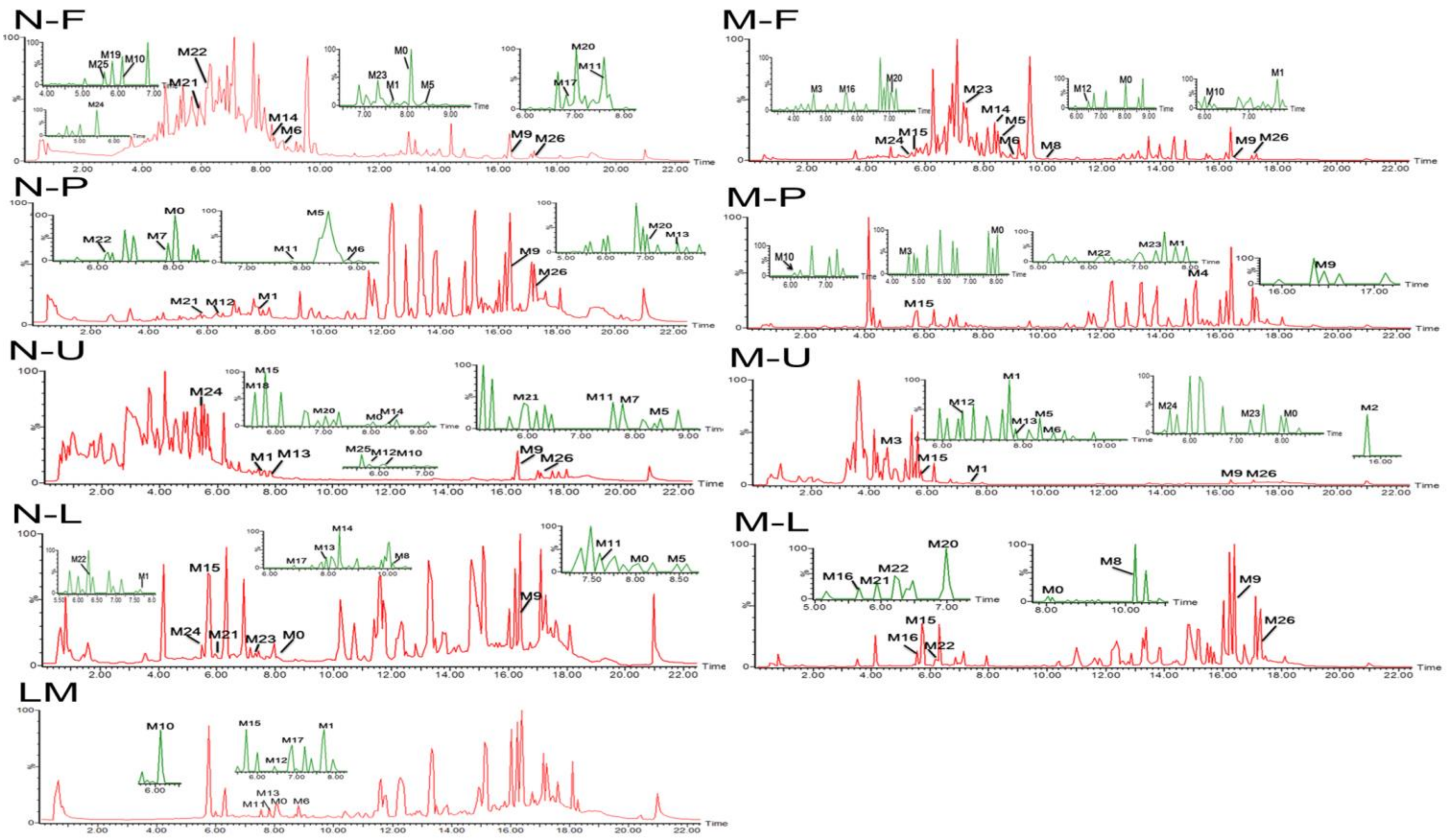

Figure S1. Typical Chromatograms of rat plasma, urine, fecal and liver, as well as liver microsomal samples. N-F: Fecal sample of normal rats; M-F: Fecal sample of model rats; N-P: Plasma sample of normal rats; M-P: Plasma sample of model rats; N-U: Urine sample of normal rats; M-U: Urine sample of model rats; N-L: Liver sample of normal rats; M-L: Liver sample of model rats; LM: Liver microsomal samples.

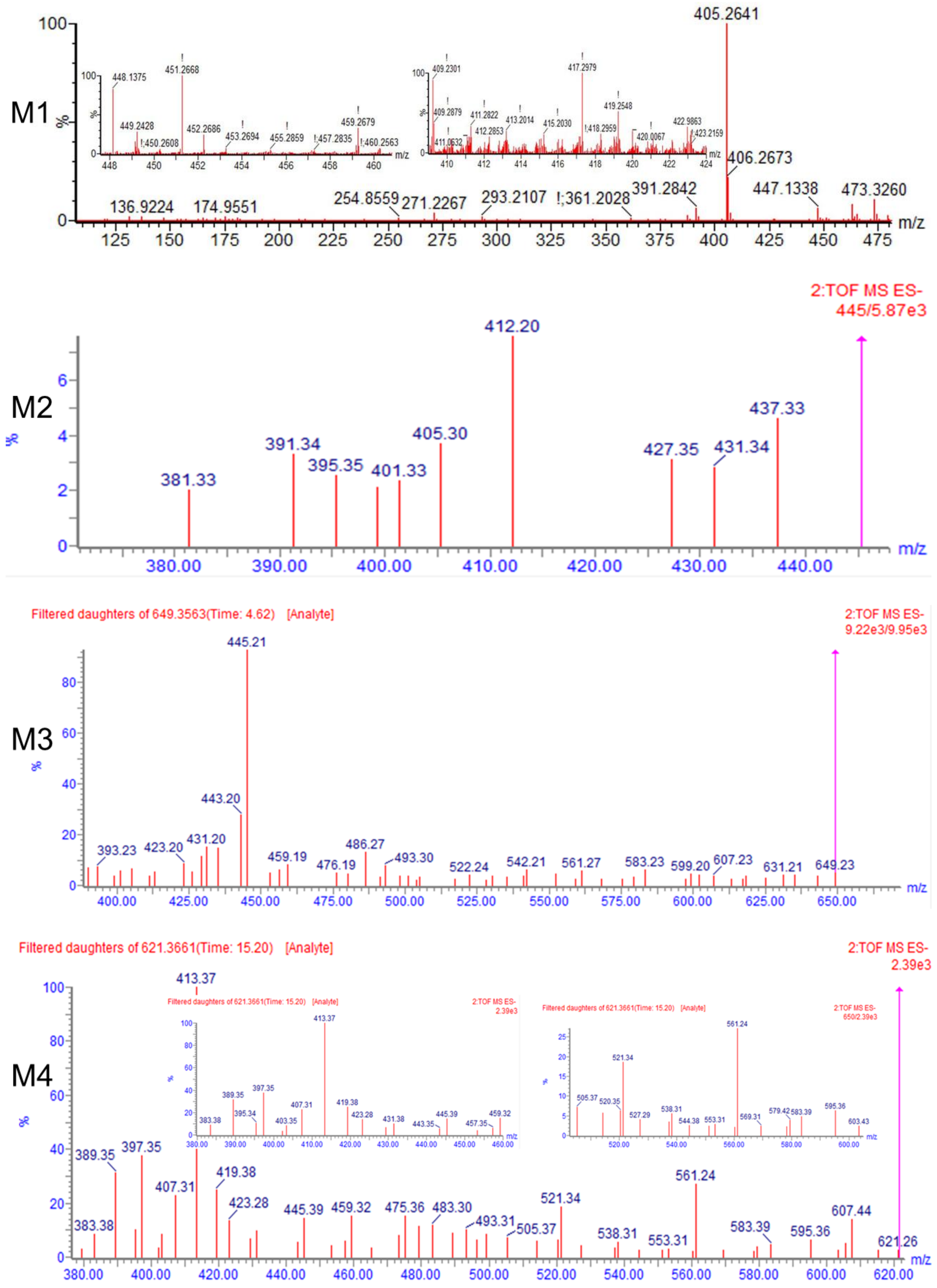

M1
M2
M3
M4
405.2641
406.2673
447.1338
473.3260
391.2842
361.2028
293.2107
271.2267
254.8559
174.9551
136.9224
m/z
2:TOF MS ES-
445/5.87e3
412.20
437.33
427.35
431.34
405.30
401.33
395.35
391.34
381.33
Filtered daughters of 649.3563(Time: 4.62) [Analyte]
2:TOF MS ES-
9.22e3/9.95e3
445.21
443.20
431.20
423.20
393.23
459.19
476.19
486.27
493.30
522.24
542.21
561.27
583.23
599.20
607.23
631.21
649.23
Filtered daughters of 621.3661(Time: 15.20) [Analyte]
2:TOF MS ES-
2.39e3
413.37
389.35
397.35
407.31
419.38
423.28
383.38
445.39
459.32
475.36
483.30
493.31
505.37
521.34
538.31
553.31
561.24
583.39
595.36
607.44
621.26

M5

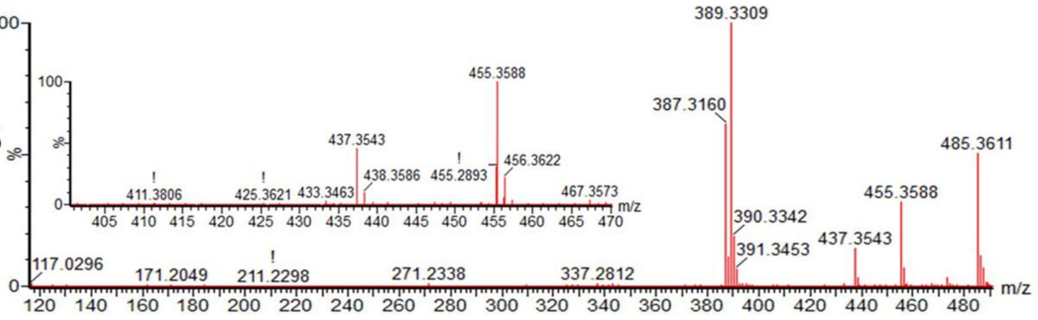
389.3309
387.3160
485.3611
455.3588
437.3543
390.3342
391.3453
117.0296
171.2049
211.2298
271.2338
337.2812
%
m/z
455.3588
437.3543
438.3586
433.3463
425.3621
411.3806
455.2893
456.3622
467.3573

M6

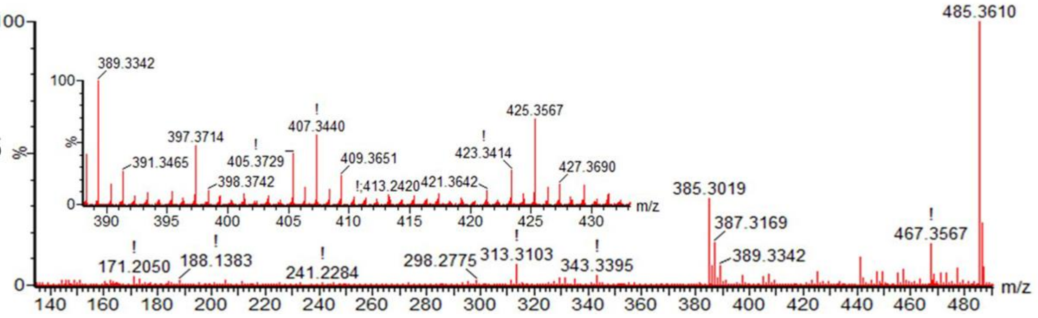
485.3610
385.3019
387.3169
389.3342
467.3567
171.2050
188.1383
241.2284
298.2775
313.3103
343.3395
%
m/z
389.3342
391.3465
397.3714
398.3742
405.3729
407.3440
409.3651
413.2420
421.3642
423.3414
425.3567
427.3690

M7

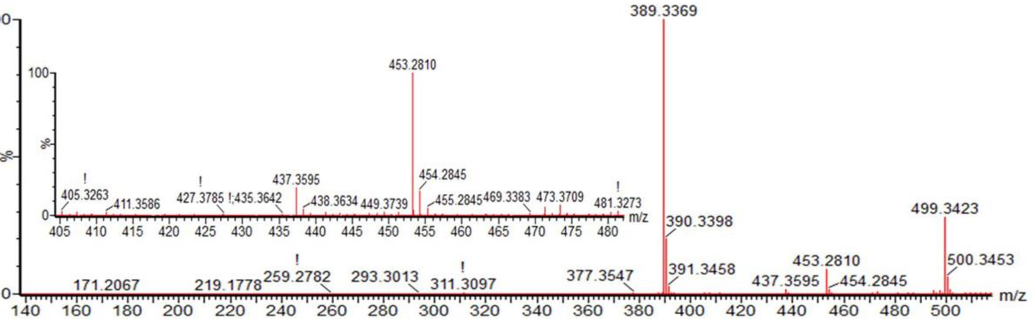
389.3369
390.3398
391.3458
499.3423
500.3453
453.2810
454.2845
437.3595
377.3547
311.3097
293.3013
259.2782
219.1778
171.2067
%
m/z
453.2810
454.2845
437.3595
438.3634
449.3739
455.2845
469.3383
473.3709
481.3273
405.3263
411.3586
427.3785
435.3642

M8

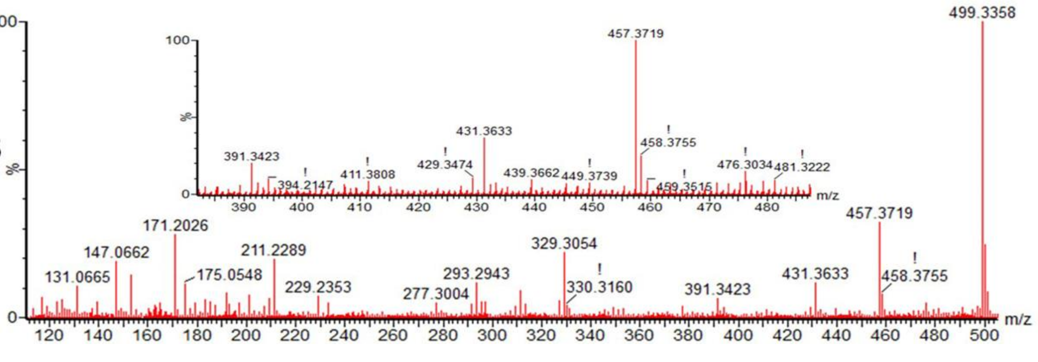
499.3358
457.3719
458.3755
431.3633
391.3423
329.3054
330.3160
293.2943
277.3004
229.2353
211.2289
175.0548
171.2026
147.0662
131.0665
%
m/z
457.3719
458.3755
431.3633
439.3662
449.3739
429.3474
411.3808
394.2147
391.3423
476.3034
481.3222
459.3515

M9

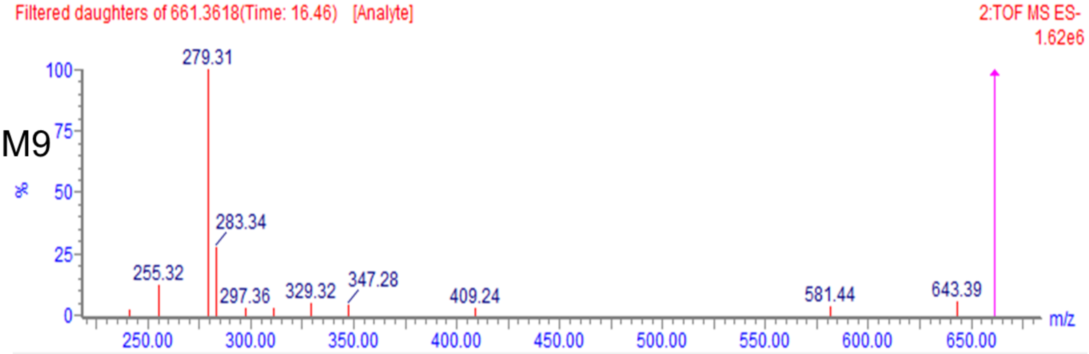

M10

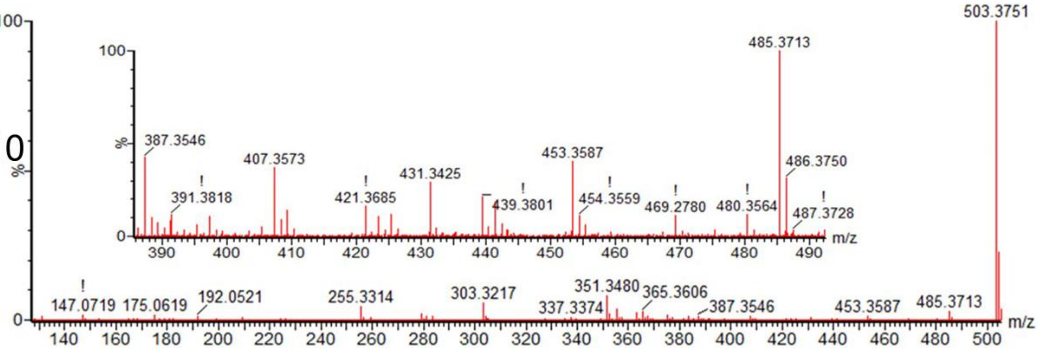

M11

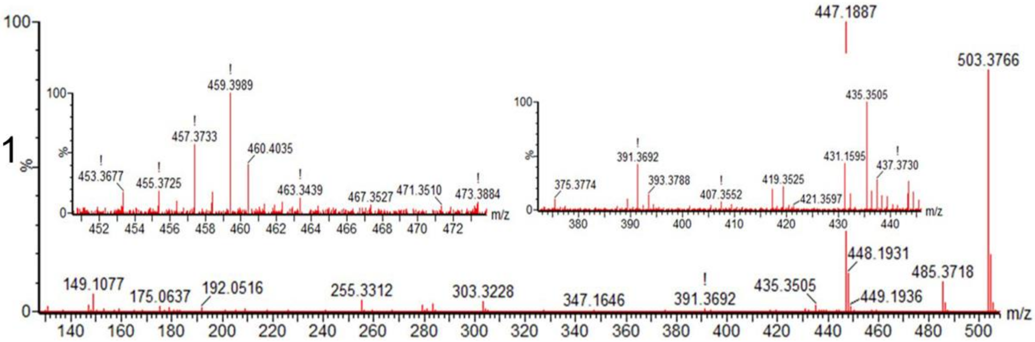

M12

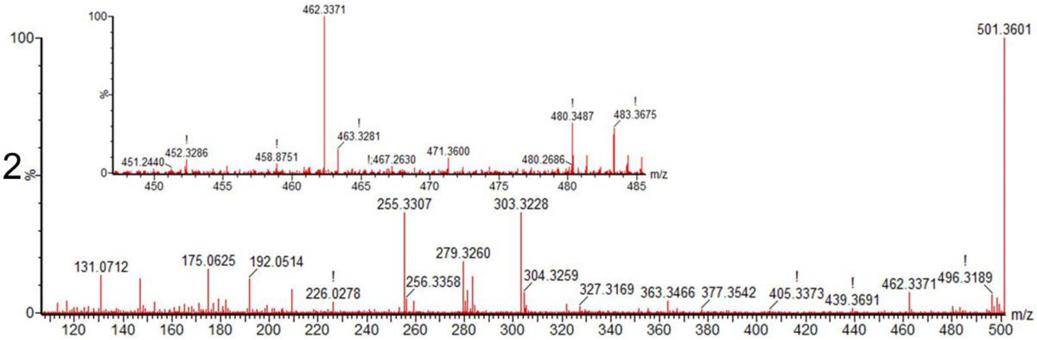

M13

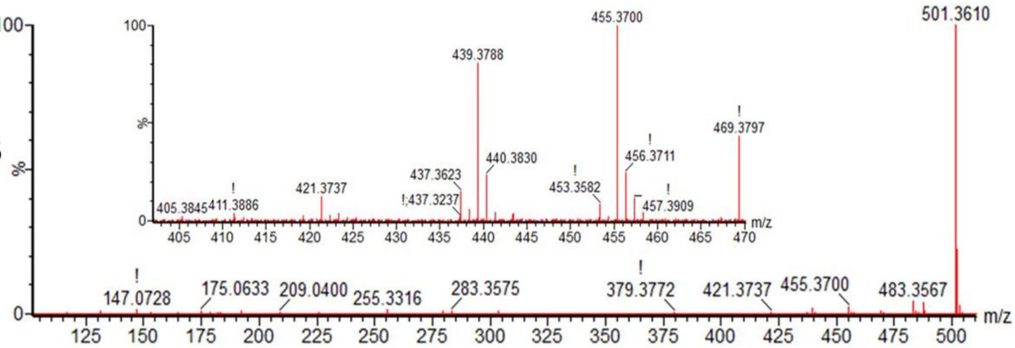

M14

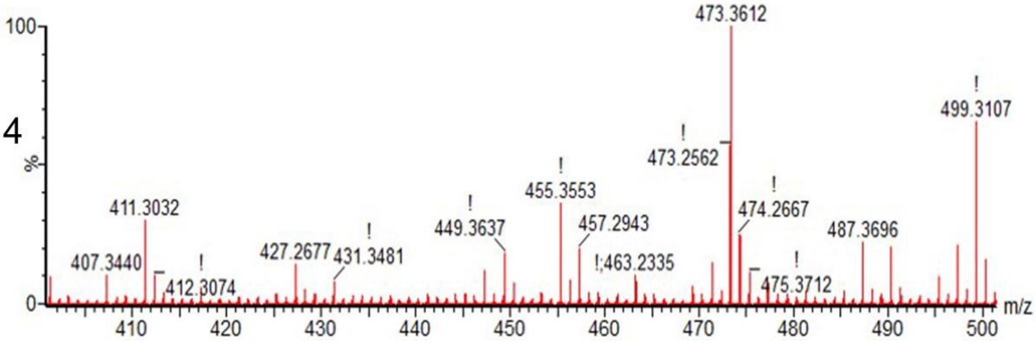

M15

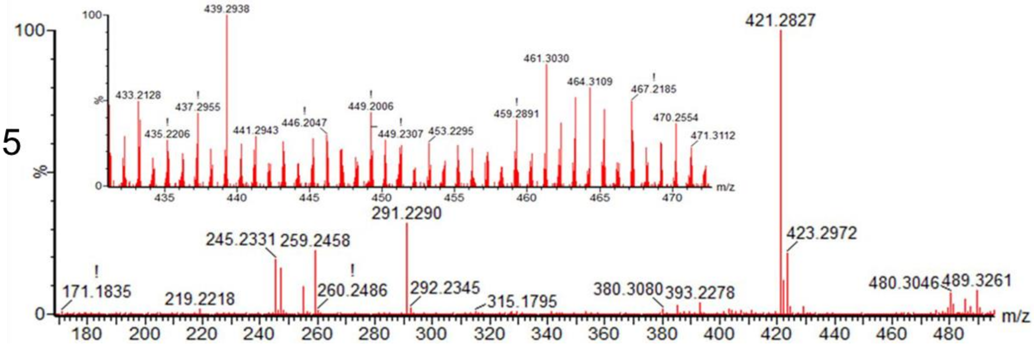

M16

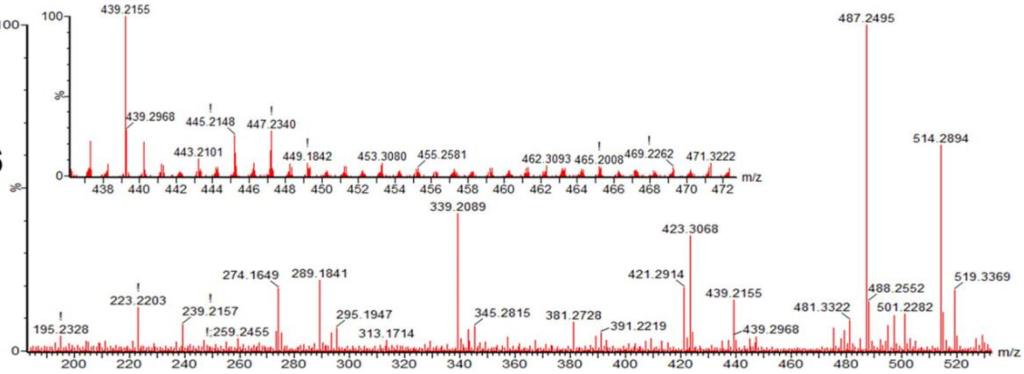

M17

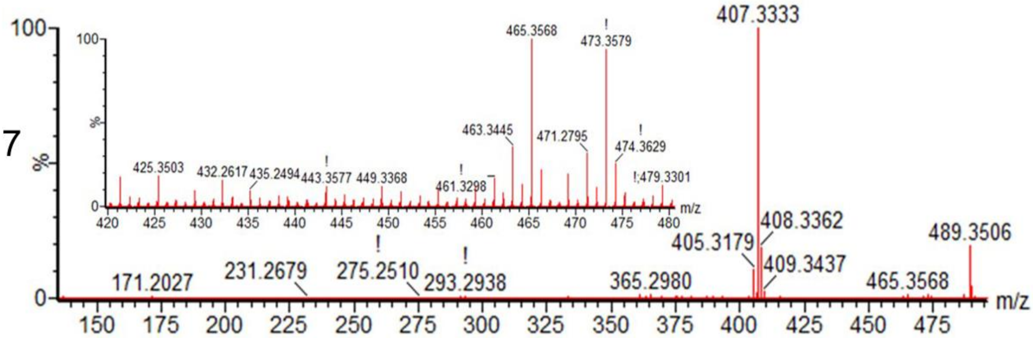

M18

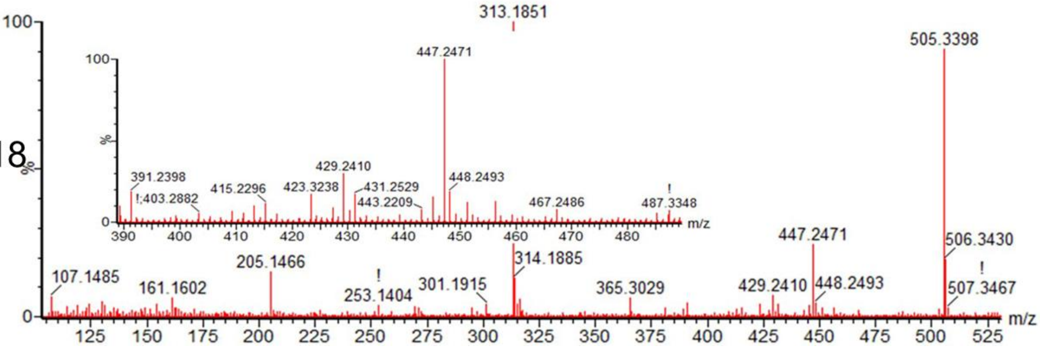

M19

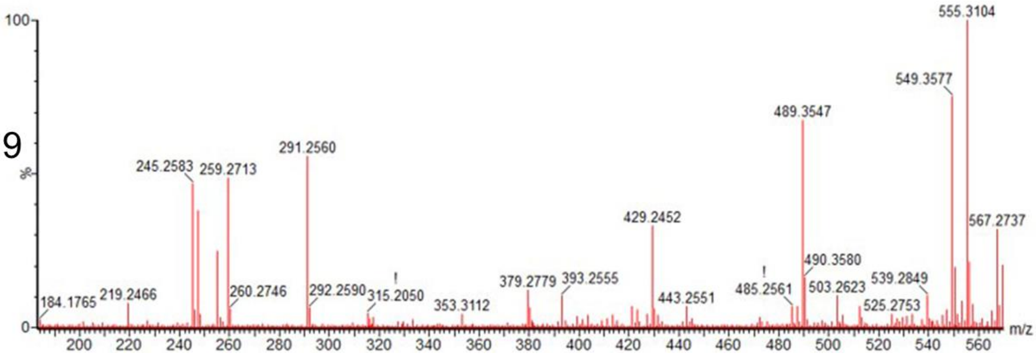

M20

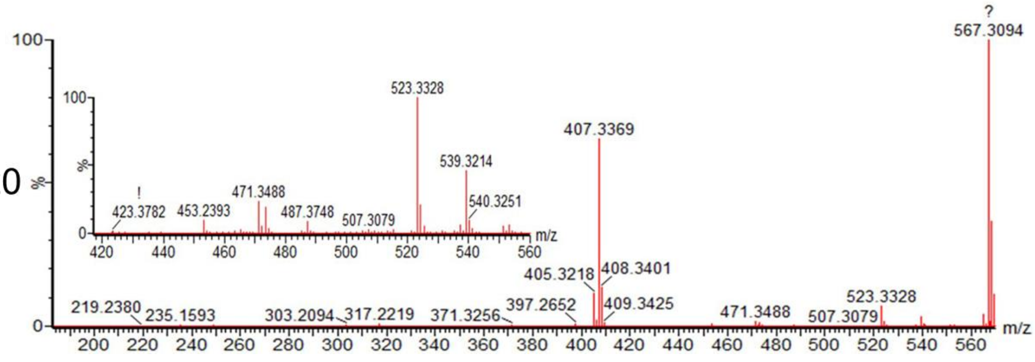

M21

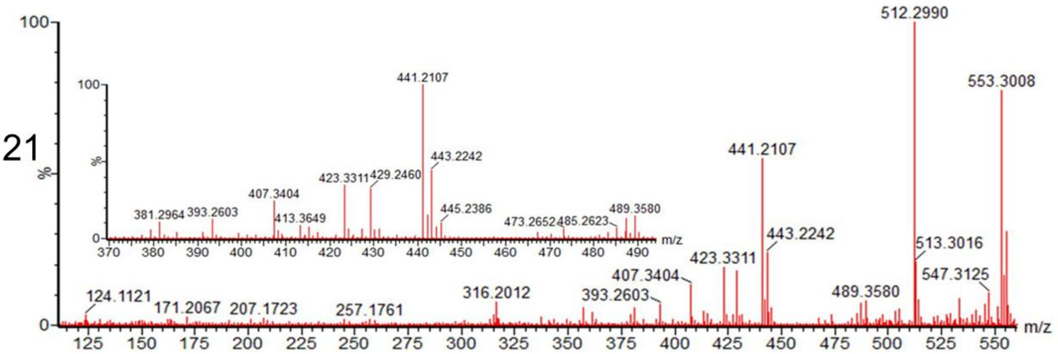

M22

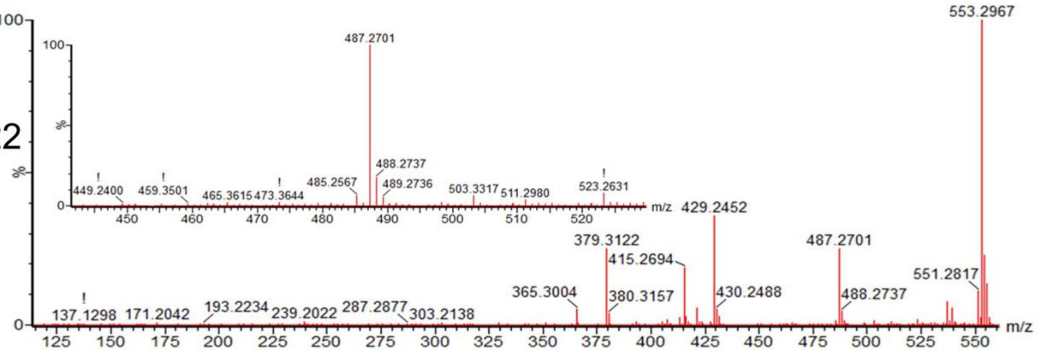

M23

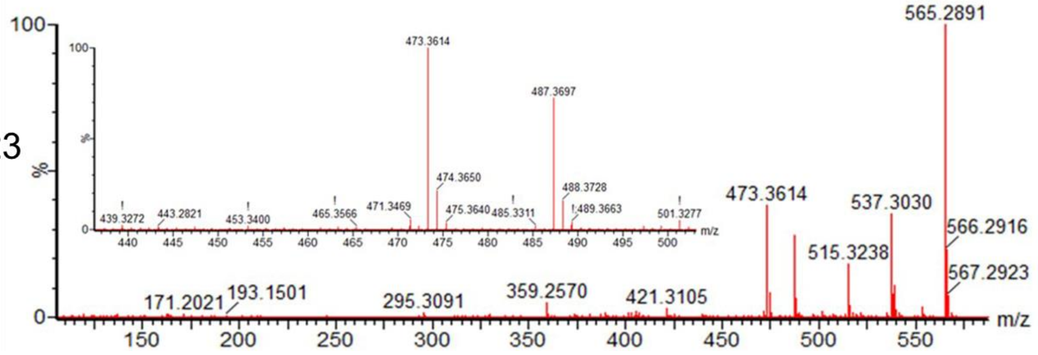

M24

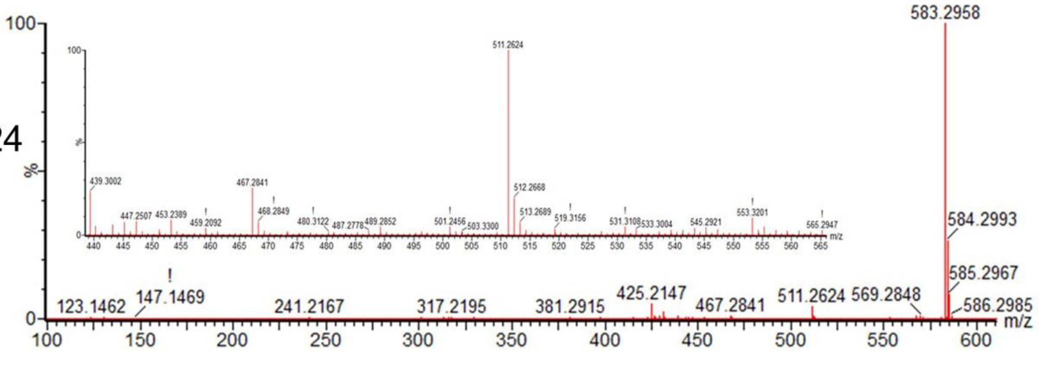

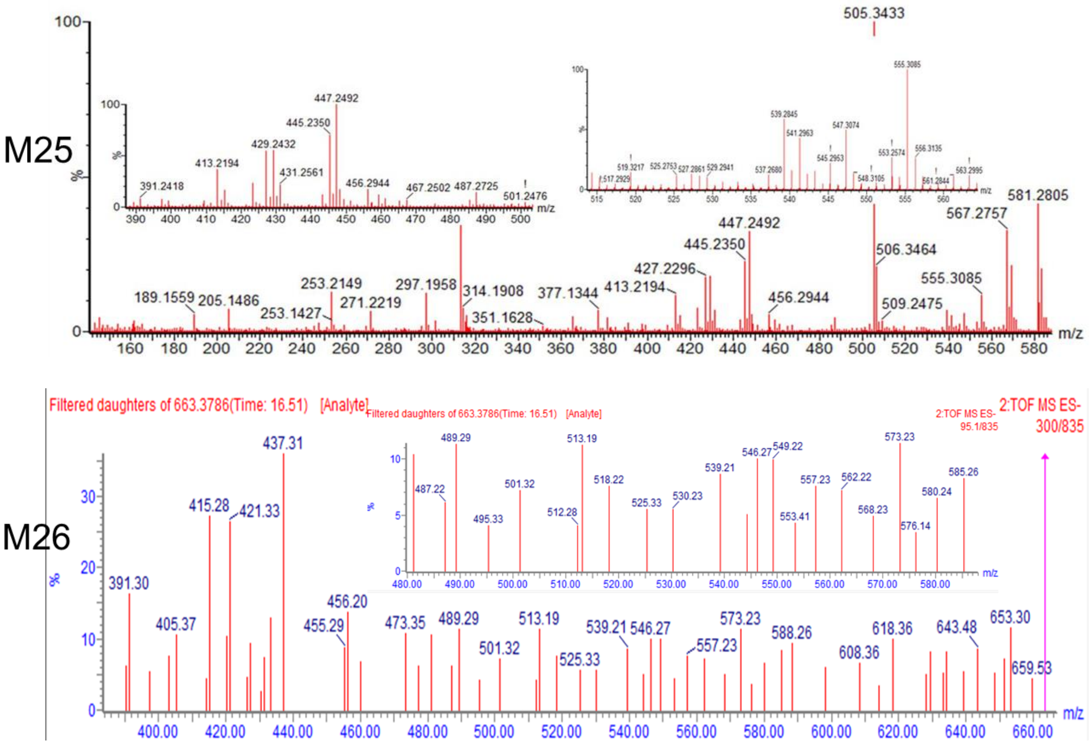

Figure S2. The MS/MS spectra of metabolites M1-M26.
